# Development and Validation of a Prediction Model for Acute Kidney Injury Among Patients With Acute Decompensated Heart Failure

**DOI:** 10.3389/fcvm.2021.719307

**Published:** 2021-11-15

**Authors:** Lei Wang, Yun-Tao Zhao

**Affiliations:** Department of Cardiology, Aerospace Center Hospital, Beijing, China

**Keywords:** acute decompensated heart failure, acute kidney injury, prediction model, B-type natriuretic peptide, acute cardiorenal syndrome

## Abstract

**Background:** Acute kidney injury is an adverse event that carries significant morbidity among patients with acute decompensated heart failure (ADHF). We planned to develop a parsimonious model that is simple enough to use in clinical practice to predict the risk of acute kidney injury (AKI) occurrence.

**Methods:** Six hundred and fifty patients with ADHF were enrolled in this study. Data for each patient were collected from medical records. We took three different approaches of variable selection to derive four multivariable logistic regression model. We selected six candidate predictors that led to a relatively stable outcome in different models to derive the final prediction model. The prediction model was verified through the use of the C-Statistics and calibration curve.

**Results:** Acute kidney injury occurred in 42.8% of the patients. Advanced age, diabetes, previous renal dysfunction, high baseline creatinine, high B-type natriuretic peptide, and hypoalbuminemia were the strongest predictors for AKI. The prediction model showed moderate discrimination C-Statistics: 0.766 (95% CI, 0.729–0.803) and good identical calibration.

**Conclusion:** In this study, we developed a prediction model and nomogram to estimate the risk of AKI among patients with ADHF. It may help clinical physicians detect AKI and manage it promptly.

## Background

Acute kidney injury is a complex systemic syndrome associated with high morbidity and mortality. In patients with acute decompensated heart failure (ADHF), the incidence and impact of acute kidney injury (AKI) have been reported mainly in subjects hospitalized with acute HF (AHF), in which the prevalence of AKI is about 20% (1). In patients with ADHF, AKI is a frequent event in which hemodynamic status, low cardiac output or congestive status, and the impact of drugs, mainly diuretics and renin–angiotensin system blockade, are relevant factors. It has been recognized that AKI is a strong independent predictor of both in-hospital and 1-year mortality (1–3).

Early detection of patients at higher risk for AKI occurrence would help physicians to plan and initiate appropriate managements to improve the renal safety of therapies, augment surveillance of cardiac and renal dysfunction, and develop renal-preserving treatments. A substantial proportion of cases of AKI are thought to be preventable with early treatment (4, 5).

Many studies have revealed the mechanism of AKI in patients with heart failure through common hemodynamic, neurohormonal, and immunological and biochemical feedback pathways (6–8). An evolution in the early diagnosis has been the discovery of novel AKI biomarkers, such as Cystatin C (9), neutrophil gelatinase-associated lipocalin (NGAL) (10, 11), kidney injury molecule 1 (12), and soluble urokinase plasminogen activator receptor (suPAR) (13). These biomarkers are not convenient for clinical acquisition and are not suitable for clinical prediction. At present, risk factors for postoperative AKI in outpatients (14), inpatients (14), critically ill patients (15–18), and surgical patients (19–21) have been analyzed, and a prediction model has been established. There is lack of a prediction model for AKI in patients with ADHF. We, therefore, derived a practical risk prediction model for AKI among patients with ADHF.

## Methods

The methods described in this article are in accordance with the transparent reporting of a multivariable prediction model for individual prognosis or diagnosis (TRIPOD) statement (22).

### Data Sources and Processing

This study was approved by the ethics committee of the Aerospace Center Hospital, Beijing, China; written informed consent was waived owing to the use of anonymous retrospective data. Data for each patient were collected from the medical records. A team of experienced cardiology clinicians reviewed and cross-checked the data. Each record was checked independently by two clinicians.

### Patient Selection

A total of 1,081 patients diagnosed with ADHF who were admitted to Aerospace Center Hospital (a tertiary hospital in Beijing, China) from January 2017 to December 2019 were retrospectively recruited. ADHF was diagnosed based on the European Society of Cardiology guidelines (23).

We excluded patients who met the following exclusion criteria: chronic kidney disease requiring regular dialysis, <18 years old, pregnant women, dementia, psychosis, length of stay <2 days, underwent surgery, and injection contrast agent in hospital. When there were two or more admissions for the same patient, only the most recent was considered. Electronic medical records were then screened, and patients were excluded if critical data for the diagnosis of AKI (such as serum creatinine levels and urine output) were missing (Supplementary Figure 1).

### Potential Predictive Variables

Consistent data for each patient were collected from the medical records. All candidate predictors were selected based on detailed literature reviews and clinical evidence within the confines of data availability (24).

Potential predictive variables included the following patient characteristics at hospital admission: clinical signs and symptoms, imaging results, laboratory findings, demographic variables, medical history, and treatment. Demographic variables collected for the study included sex, age, height, and weight. Medical history included diabetes, hypertension, coronary artery disease, previous heart failure, atrial fibrillation, previous renal dysfunction, cerebral infarction, cancer, and cirrhosis. Clinical signs and symptoms included categorical and continuous variables: New York Heart Association (NYHA) functional class, heart rate, systolic blood pressure, diastolic blood pressure, rales (>1/2 lung fields), jugular venous distension, and peripheral edema. Imaging results included left ventricular ejection fraction (LVEF) by two-dimensional transthoracic echocardiography. Laboratory findings included B-type natriuretic peptide, hemoglobin, hematocrit, C-reactive protein, alanine aminotransferase, total bilirubin, blood urea nitrogen, creatinine, albumin, serum sodium, serum potassium, uric acid, and glucose. We recorded the baselines of these tests with the first value being within 2 days of onset admission. Treatment included aldosterone antagonists, loop diuretic, angiotensin-converting enzyme inhibitors/angiotensin receptor blockers (ACE-Is/ARBs), beta-blockers, anticoagulants, aspirin, non-steroidal anti-inflammatory drugs (NSAIDs), vasopressor use, intra-aortic balloon pump (IABP), and mechanical ventilator. These detailed and specific definitions are listed in Supplementary Table 1.

### Definition of AKI

The outcome was the occurrence of AKI during the hospital stay of the patients, according to the Kidney Disease: Improving Global Outcomes (KDIGO) guidelines (25). Any patient meeting criteria for stage 1 or higher, based on either serum creatinine level or urine output, was considered to have AKI. (Criteria for stage 1 : serum creatinine, 1.5–1.9 times baseline or ≥26.5 μmol/L increase; urine output, <0.5 ml/kg/h for 6–12 h), we placed that patient into the AKI group. The creatinine measurement obtained the first value being within 2 days of onset admission to the hospital was used as the baseline value for all analyses.

### Sample Size

We originally considered events per variables (EPV) ratio between 5 and 10 acceptable, with EPV of 10 as the optimal number to minimize overfitting of the regression model. According to this rule, we needed 60 ADHF inpatient AKI occurrence to evaluate six candidate predictors. Assuming that the prevalence of acute kidney injury was 20% among patients with ADHF (26), a total sample size of at least 300 would suffice. To ensure an adequate number of events, we decided to collect data of at least 600 individuals.

### Missing Data

Before data analysis, predictor variables were inspected for missing values. Among the predictors, the proportion of missing data was 0.3–6 %. To include these data from the analyses, we imputed missing data by multiple imputations by chained equations, using the mice package for R, in which predictive mean matching is embedded with the cases (k) = 5 default. Baseline clinical characteristics before and after imputation are listed in Supplementary Table 2.

### Statistical Analysis

Data are presented as frequencies (percentages) for categorical variables and mean (standard deviation) or medians (interquartile ranges [IQRs]) for continuous variables. Means for continuous variables were compared by *t*-tests when the data were normally distributed; otherwise, the Mann–Whitney U test was performed. Proportions for categorical variables were compared by the χ^2^ test, although the Fisher exact probability test was performed when the data were limited. The significance level of the above statistical analyses was set as α = 0.05, and *P* < 0.05 (two-tailed) was considered statistically significant.

### Variable Selection and Model Development

Different models will be constructed based on different variables. Some variables, which may be closely related to the true world, will lead to a relatively stable outcome even in different models (24, 27). To ensure the robustness and validity of the prediction model, we took three different approaches of variable selection to derive four multivariable logistic regression models: Akaike information criterion (AIC)-based stepwise (model A), multifractional polynomial (MFP) (model B), least absolute shrinkage and selection operator (LASSO) regression (model C and model D according to tuning parameter lambda.1se and lambda.min, respectively).

If the relationship between the variable and the outcome is not linear, the variable can be transformed with the MFP model (24). LASSO regularization was used for variable selection, and logistic regression was used to estimate the association of risk factors and AKI. LASSO regression is a compression estimation that is used to deal with the collinearity between covariates. When there are several collinear predictors, LASSO selects only one and ignores the others or zeroes out some regression coefficients. Multicollinearity was tested using the variance inflation factor (VIF) method, with a VIF ≥ 10 indicating the presence of several multicollinearities.

The analysis results and clinical reasons, sample size, and statistical power should be considered at the same time. We evaluated the C-Statistics and calibration curve of each model, evaluated the variables of the four models, and selected six most stable predictors based on their clinical significance (28).

Finally, we selected six candidate predictors to derive the prediction model, and built a nomogram based on the results of a logistic regression model. If the relationship between the variable and the outcome is not linear, the variable can be transformed by the MFP model. We evaluated the C-Statistics of the two models.

### Model Validation

We carried out internal validation of the model development process using a bootstrap resampling process (500 bootstrap samples per model) to provide an unbiased estimate of model performance (24). Then, the prediction model was verified through the use of the C-Statistics, calibration curve (29), and Decision Curve Analysis (28).

### Sensitivity Analysis

Given the heterogeneity of sex and age, to investigate whether the predictive strength of nomogram would change by sex or age, we investigated the interaction between creatinine and sex or age with the Wald test. We considered a two-sided *P* value of < 0.05 to be statistically significant. If there is an interaction between sex or age and creatinine, we divided the cohort into two subgroups based on sex and age (threshold of 65 years) to investigate whether the prediction model performed equally well.

### Statistical Analysis Software

Data were analyzed with the use of the statistical packages R (The R Foundation; http://www.r-project.org; version 3.4.3) and Empower (R) (version 3.0; www.empowerstats.com, X&Y solutions, Inc., Boston, MA, United States;).

## Results

### Statistical Analysis

In total, 650 patients with ADHF were enrolled in this study, and 278 had AKI, meaning that the incidence of AKI was 42.8%. With the mean age of 74.8 years, 298 (45.8%) of the patients were male. Compared with non-AKI patients, patients with AKI were significantly older (*P* < 0.05). Supplementary Table 3 shows a comparison of the characteristics of patients enrolled in the study. The proportions of medical history (diabetes, coronary artery disease, previous congestive heart failure, atrial fibrillation and previous renal dysfunction) and laboratory findings (B-type natriuretic peptide, hemoglobin, hematocrit, alanine aminotransferase, blood urea nitrogen, creatinine, and albumin) were significantly different (P < 0.05).

### Variable Selection and Model Development

We developed model A of variable selection by AIC-based stepwise that consisted of 15 variables. We further developed model B by MFP-selected variables that also consisted of 15 variables (B-type natriuretic peptide divided by 1,000, age, creatinine, albumin divided by 100, and C-reactive protein divided by 10).

Based on LASSO analysis (Supplementary Figure 2), we identified model C and model D that consisted of 7 and 19 variables with the tuning parameters lambda.1se and lambda.min, respectively (Supplementary Table 4).

We evaluated the C-Statistics (Supplementary Table 5) and a calibration curve of each model (Supplementary Figure 3).

**Figure 1:**
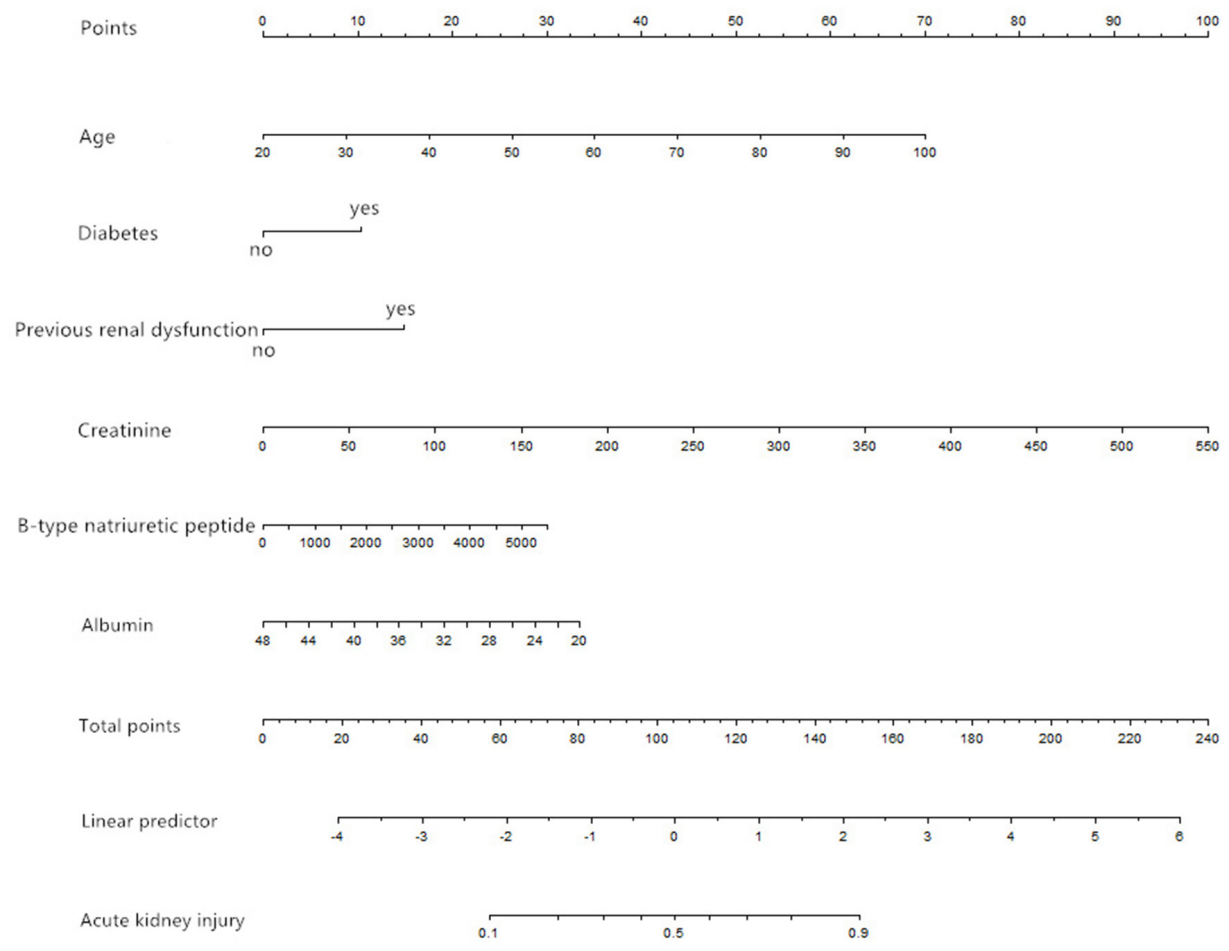
Nomogram based on the results of a logistic regression model (age in units of years, creatinine in units of μmol/L, B-type natriuretic peptide in units of pg/ml, albumin in units of g/L).

We selected the six most stable candidate predictors to derive the prediction model, and built a nomogram based on the results of a logistic regression model (Figure 1).

A risk score was calculated for each patient using a formula derived from the expression levels of the six variables weighted by their regression coefficients: Risk score = −3.02492 + 0.04100 × age (years) + 0.48031 × (diabetes = 1) + 0.69499 × (previous renal dysfunction = 1) + 0.00851 × creatinine (μmol/L) + 0.00026 × B-type natriuretic peptide (pg/ml) −0.056 × albumin (g/L).

As the relationship between four variable and the outcome is not linear, the variable was transformed by the MFP (B-type natriuretic peptide divided by 1,000, age, creatinine, albumin divided by 100). We found that two models had similar prediction performance in terms of C-Statistics (MFP model, C-Statistics: 0.766 [95% CI, 0.729–0.803]; prediction model, C-Statistics: 0.766 [95% CI, 0.729–0.803]), so we finally selected the prediction model for validation, taking into consideration the clinical significance, in which there is no need for variable divided by 100 or 1,000.

### Model Validation

**Figure 2:**
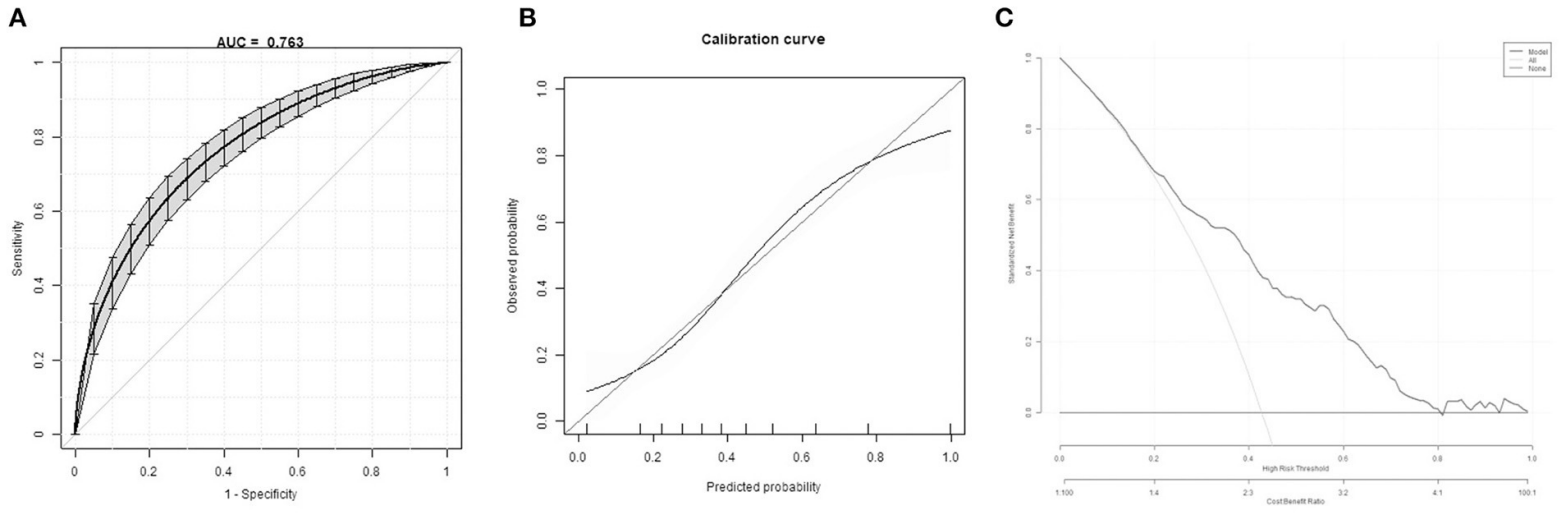
Model internal validation. **(A)** Discrimination of the prediction model by the bootstrap method. **(B)** Calibration plots of the model by the bootstrap method. **(C)** Decision curve of the model.

The bootstrap method showed a moderate discriminative ability of prediction model (C-Statistics: 0.763 [95% CI, 0.73–0.803] (Figure 2A). Calibration plots of the model by the bootstrap method showed good performance at most (Figure 2B). Decision curve analysis showed moderate clinical effectiveness of the models (Figure 2C).

### Sensitivity Analysis

We did not find the interaction between sex and creatinine (*P* = 0.89, crude, *P* = 0.41, adjusted). We found the interaction between age (threshold of 65 years) and creatinine (*P* = 0.13, crude, *P* = 0.01, adjusted). Given the heterogeneity of age, we divided the cohort into two subgroups (122 younger and 528 older patients) based on the age threshold of 65 years to investigate whether the prediction model performed equally well in the older and younger patients. The discrimination of the prediction model was consistent for the younger subgroup (C-Statistics: 0.825 [95% CI, 0.747–0.903]) and the older subgroup (C-Statistics: 0.748 [95% CI, 0.707–0.79]). The calibration curve indicated that the prediction model was similar calibrated in subgroups (Supplementary Figure 4).

### Nomogram Interpretation

The point in Figure 1 is a selected scoring standard or scale. For each independent variable, a straight line perpendicular to the axis of points (through a ruler) is made at that point, and the intersection point represents the score under the value of the independent variable. For example, age at 60 means 35 points; creatinine at 150 means 27.5 points. The corresponding points of these independent variables of each patient can be calculated in total. We can get total points, which will locate to the axis with a perpendicular line. This will indicate the risk for AKI occurrence of this patient.

## Discussion

Heart and kidney interactions are complex and a subject of immense clinical and scientific interest and debate. The coexistence of acute cardiac and renal dysfunction, termed as acute cardiorenal syndrome, has been shown to correlate with increased mortality and all manners of adverse outcomes (6). Currently, there is no effective practical tool for estimating the likelihood of AKI occurrence after ADHF. In this study, we developed a risk score and nomogram to estimate the risk of AKI among patients with ADHF. This risk score system in the study is easy and convenient to apply for early-stage AKI prediction. It may help clinical physicians detect AKI and manage it promptly.

Based on different regressions, we found six most stable risk factors of AKI occurrence in this retrospective study, namely, advanced age, diabetes, previous renal dysfunction, high baseline creatinine, high B-type natriuretic peptide, and hypoalbuminemia. Some established risk factors are advanced age, chronic heart disease, and previous renal dysfunction (30). Advanced age, diabetes, and high creatinine (baseline renal function) at admission were also found in other AKI prediction models (31). Recent research has identified several non-traditional risk factors for AKI, which clinicians caring for acutely ill patients should be aware of. The B-type natriuretic peptide has emerged as an important tool for the diagnosis and risk stratification of patients with heart failure. Our study and Hogenhuis et al. found that renal dysfunction is independently associated with B-type natriuretic peptide levels in patients with heart failure (32). Patel et al. found that B-type natriuretic peptide level is associated with postoperative AKI in high-risk patients undergoing cardiac surgery (33). Low serum albumin is common in patients with heart failure and is associated with increased mortality (34, 35). Renal dysfunction may be the main pathophysiological mechanism underlying hypoalbuminemia in patients with heart failure. The association between hypoalbuminemia and development of AKI and subsequent morbidity/mortality can also be regarded as confirmed (36). B-type natriuretic peptide and hypoalbuminemia reflect the interaction between the heart and the kidney.

The discussion on variable screening has been going on for a long time. We found out the most stable factor for the outcome from results based on C-Statistics and plotted the calibration curve of four models by three different approaches. The analysis results and clinical reasons, sample size, and statistical power were considered at the same time. A prediction model is constructed with traditional clinical features and laboratory test results. The predictive variables in the nomogram model are convenient for clinical acquisition, and the construction of these models is feasible. These parsimonious models will be sufficiently stable for application (24). Sometimes algorithms of machine learning are used to build models, and most of these models are non-parametric. Because there are no parameters like regression coefficients, the clinical interpretation of such nonparametric models is difficult (24). Therefore, we did not select machine learning for variable filtering. The study took many steps to minimize potential bias. For instance, we excluded repeated admissions to ensure that all cases in the final cohort were independent from each other (37).

### Limitations

In addition to these findings, some limitations to our study should be addressed. First, this is an observational study that cannot directly draw causal conclusions. Second, this study excluded patients of injection contrast agent and surgery, so this population could not be extrapolated. Third, some pieces of information, such as a history of myocardial infarction and etiology of heart failure, were not inputted into the analysis, and this may need to be investigated in future research. Finally, as this study was based on a single center in China, it will inevitably have sample selection bias. We lack external validation of the study. As such, prospective validation to examine model stability, reproducibility, and external validity in independent samples is needed.

## Conclusion

Acute kidney injury is an adverse event that carries significant morbidity among patients with ADHF. Currently, there is no effective practical tool for estimating the likelihood of AKI occurrence after ADHF. In this study, we developed a risk score and nomogram to estimate the risk of AKI among patients with ADHF. It may help clinical physicians detect AKI and manage it promptly.

## Data Availability Statement

The original contributions presented in the study are included in the article/Supplementary Materials, further inquiries can be directed to the corresponding author.

## Ethics Statement

The studies involving human participants were reviewed and approved by 航天中心医院医学伦理委员会. Written informed consent for participation was not required for this study in accordance with the national legislation and the institutional requirements.

## Author Contributions

Y-TZ and LW made contributions to data collection, drafted the manuscript, and analysis and interpretation of the data. All authors contributed to the article and approved the submitted version.

## Conflict of Interest

The authors declare that the research was conducted in the absence of any commercial or financial relationships that could be construed as a potential conflict of interest.

## Publisher's Note

All claims expressed in this article are solely those of the authors and do not necessarily represent those of their affiliated organizations, or those of the publisher, the editors and the reviewers. Any product that may be evaluated in this article, or claim that may be made by its manufacturer, is not guaranteed or endorsed by the publisher.
