# Supplementary material for: Development and Validation of a Prediction Model for Acute Kidney Injury Among Patients With Acute Decompensated Heart Failure
Source: Front Cardiovasc Med. 2021 Nov 15;8:719307. doi: 10.3389/fcvm.2021.719307 (PMC8634389; doi:10.3389/fcvm.2021.719307)
Supplement: Supplementary file 1 [file Table_1.DOC]

Supplemental Material

Table S1. Definition of variables.

| Variables | Method of assessment and grading | Reference |
| --- | --- | --- |
| **Demographic information** |  |  |
| Sex | Female  Male | female vs male |
| Age | Assessed at admission | in units of years |
| Height | Assessed at admission | in units of cm |
| Weight | Assessed at admission | in units of Kg |
| **Medical History** |  |  |
| Diabetes | Diabetes mellitus, Type 1 or 2 diabetes, taking hypoglycemic medications | absence vs presence |
| Hypertension | Hypertension, primary or secondary Hypertension, hypertension grade 1, 2 or 3 | absence vs presence |
| Coronary artery disease | Coronary artery disease, angina, myocardial infarction, ischemic heart disease, coronary artery stent implantation | absence vs presence |
| Previous heart failure | Heart failure, systolic heart failure, diastolic heart failure, cardiac dysfunction, heart failure unspecified | absence vs presence |
| Atrial fibrillation | Atrial fibrillation, paroxysmal or persistent atrial fibrillation | absence vs presence |
| Previous renal dysfunction | Renal dysfunction, renal failure, renal failure unspecified | absence vs presence |
| Cerebral infarction | Cerebral infarction, cerebral stroke | absence vs presence |
| Cancer | Cancer, tumors of the organs or blood systems | absence vs presence |
| Cirrhosis | Liver cirrhosis, compensatory or decompensated cirrhosis | absence vs presence |
| **Clinical information** |  |  |
| NYHA classification | Assessed according to the New York Heart Association (NYHA) Functional Classification  I: Cardiac disease, but no symptoms and no limitation in ordinary physical activity, e.g. no  shortness of breath when walking, climbing stairs etc.  II: Mild symptoms (mild shortness of breath and/or angina) and slight limitation during  ordinary activity.  III: Marked limitation in activity due to symptoms, even during less-than-ordinary activity,  e.g. walking short distances (20–100 m); comfortable only at rest.  IV: Severe limitations. Experiences symptoms even while at rest. | II vs III vs IV |
| Heart rate | Assessed at admission | in units of beats / minute |
| Systolic blood pressure | Assessed at admission | in units of mmHg |
| Diastolic blood pressure | Assessed at admission | in units of mmHg |
| Rales (>1/2 lung fields) | Assessed at admission | absence vs presence |
| Jugular venous distension | Assessed at admission | absence vs presence |
| Peripheral edema | Assessed at admission | absence vs presence |
| **Imaging results** |  |  |
| LVEF | The first value being within 2 days of onset admission by two-dimensional transthoracic echocardiography | in units of % |
| **Laboratory findings** |  |  |
| B-type natriuretic peptide | The first value being within 2 days of onset admission | in units of pg/ml |
| Hemoglobin | The first value being within 2 days of onset admission | in units of g/L |
| Hematocrit | The first value being within 2 days of onset admission | in units of % |
| C-reactive protein | The first value being within 2 days of onset admission | in units of mg/L |
| Alanine aminotransferase | The first value being within 2 days of onset admission | in units of IU/L |
| Total bilirubin | The first value being within 2 days of onset admission | in units of μmol/L |
| Blood urea nitrogen | The first value being within 2 days of onset admission | in units of mmol/L |
| Creatinine | The first value being within 2 days of onset admission | in units of μmol/L |
| Albumin | The first value being within 2 days of onset admission | in units of g/L |
| Serum sodium | The first value being within 2 days of onset admission | in units of mmol/L |
| Serum potassium | The first value being within 2 days of onset admission | in units of mmol/L |
| Uric acid | The first value being within 2 days of onset admission | in units of μmol/L |
| Glucose | The first value being within 2 days of onset admission | in units of mmol/L |
| **Treatment** |  |  |
| Aldosterone antagonists | Within 7 days of onset admission | Used or not used |
| Loop diuretic | Within 7 days of onset admission | Used or not used |
| ACE-Is/ARBs | Within 7 days of onset admission | Used or not used |
| Beta-blockers | Within 7 days of onset admission | Used or not used |
| Anticoagulants | Within 7 days of onset admission | Used or not used |
| Aspirin | Within 7 days of onset admission | Used or not used |
| NSAIDs | Within 7 days of onset admission | Used or not used |
| Vasopressor | Within 7 days of onset admission | Used or not used |
| IABP | Within 7 days of onset admission | Used or not used |
| Mechanical ventilator | Within 7 days of onset admission | Used or not used |

Variables were chosen based on a previous literature review of risk factors for AKI and additional known risk factors for AKI from other causes.

History of diabetes or hypertension recorded using diagnosis codes (International Classification of Diseases, Ninth Revision, Clinical Modification.

Abbreviations:

NYHA, New York Heart Association

LVEF, left ventricular ejection fraction

ACE-Is/ARBs, angiotensin-converting enzyme inhibitors / Angiotensin Receptor Blockers

NSAIDs, Non-Steroidal Antiinflammatory Drugs

IABP, intra-aortic ballon pump

## Table S2. Baseline characteristics of 650 patients: pre-imputation and post-imputation.

| Variables | Pre-imputation | Post-imputation |
| --- | --- | --- |
| Sex, Male | 298 (45.8%) | 298 (45.8%) |
| Age, years | 74.8 (12.8) | 74.8 (12.8) |
| Height(cm) | 163.8 (8.1) | 163.8 (8.1) |
| Weight(kg) | 65.7 (13.4) | 65.7 (13.4) |
| Diabetes | 350 (53.8%) | 350 (53.8%) |
| Hypertension | 448 (68.9%) | 448 (68.9%) |
| Coronary artery disease | 435 (66.9%) | 435 (66.9%) |
| Previous heart failure | 184 (28.3%) | 184 (28.3%) |
| Atrial fibrillation | 287 (44.2%) | 287 (44.2%) |
| Previous renal dysfunction | 108 (16.6%) | 108 (16.6%) |
| Cerebral infarction | 110 (16.9%) | 110 (16.9%) |
| Cancer | 58 (8.9%) | 58 (8.9%) |
| Cirrhosis | 7 (1.1%) | 7 (1.1%) |
| NYHA classification |  |  |
| II | 167 (25.7%) | 167 (25.7%) |
| III | 299 (46.0%) | 299 (46.0%) |
| IV | 184 (28.3%) | 184 (28.3%) |
| Heart rate (beats/min) | 91.8 (23.3) | 91.8 (23.3) |
| Systolic blood pressure (mmHg) | 132.2 (24.8) | 132.2 (24.8) |
| Diastolic blood pressure (mmHg) | 74.2 (17.0) | 74.2 (17.0) |
| Rales (>1/2 lung fields) | 311 (47.8%) | 311 (47.8%) |
| Jugular venous distension | 131 (20.2%) | 131 (20.2%) |
| Peripheral edema | 409 (62.9%) | 409 (62.9%) |
| LVEF (%) | 51.1 (12.2) | 51.0 (12.2) |
| Missing | 1(0.2%) |  |
| B-type natriuretic peptide (pg/ml) | 824.5 (366.0-1666.0) | 831.0 (359.8-1701.8) |
| Missing | 20(3.1%) |  |
| Hemoglobin (g/L) | 117.8 (24.6) | 117.8 (24.6) |
| Hematocrit (%) | 0.4 (0.1) | 0.4 (0.1) |
| C-reactive protein (mg/L) | 9.4 (4.1-24.8) | 9.2 (4.0-24.6) |
| Missing | 39(6.0%) |  |
| Alanine aminotransferase (IU/L) | 16.4 (11.3-27.8) | 16.3 (11.2-27.8) |
| Missing | 2(0.3%) |  |
| Total bilirubin | 14.2 (9.8-21.8) | 14.2 (9.8-21.7) |
| Missing | 2(0.3%) |  |
| Blood urea nitrogen (mmol/L) | 8.2 (6.0-11.6) | 8.2 (6.0-11.6) |
| Creatinine (μmol/L) | 92.7 (73.9-126.7) | 92.7 (73.9-126.7) |
| Albumin (g/L) | 35.9 (4.5) | 35.9 (4.5) |
| Sodium (mmol/L) | 138.3 (5.4) | 138.3 (5.4) |
| Potassium (mmol/L) | 4.3 (0.6) | 4.3 (0.6) |
| Uric acid (μmol/L) | 401.0 (312.0-521.8) | 401.1 (312.1-522.1) |
| Missing | 1(0.2%) |  |
| Glucose (mmol/L) | 8.4 (3.9) | 8.4 (3.9) |
| Aldosterone antagonists | 520 (80.0%) | 520 (80.0%) |
| Loop diuretic | 623 (95.8%) | 623 (95.8%) |
| ACE-Is/ARBs | 259 (39.8%) | 259 (39.8%) |
| Beta-blockers | 474 (72.9%) | 474 (72.9%) |
| Anticoagulants | 221 (34.0%) | 221 (34.0%) |
| Aspirin | 271 (41.7%) | 271 (41.7%) |
| NSAIDs | 38 (5.8%) | 38 (5.8%) |
| Vasopressor | 60 (9.2%) | 60 (9.2%) |
| IABP | 10 (1.5%) | 10 (1.5%) |
| Mechanical ventilator | 128 (19.7%) | 128 (19.7%) |

Data are presented as frequencies (percentages) or mean (SD) or median (IQR)

Abbreviations:

IQR, interquartile range; SD, standard deviation

NYHA, New York Heart Association

LVEF, left ventricular ejection fraction

ACE-Is/ARBs, angiotensin converting enzyme inhibitors / Angiotensin Receptor Blockers

NSAIDs, Non-Steroidal Antiinflammatory Drugs

IABP, intra-aortic ballon pump

Table S3 Comparison of the characteristics between the AKI and non-AKI groups

| Variables | non-AKI | AKI | P-value |
| --- | --- | --- | --- |
| N | 372 (57.2%) | 278 (42.8%) |  |
| Sex, Male | 171 (46.0%) | 127 (45.7%) | 0.943 |
| Age, years | 72.5 (13.6) | 77.9 (10.9) | <0.001 |
| Height(cm) | 163.9 (8.2) | 163.7 (8.1) | 0.614 |
| Weight(kg) | 66.5 (14.0) | 64.6 (12.3) | 0.185 |
| Diabetes | 181 (48.7%) | 169 (60.8%) | 0.002 |
| Hypertension | 245 (65.9%) | 203 (73.0%) | 0.051 |
| Coronary artery disease | 225 (60.5%) | 210 (75.5%) | <0.001 |
| Previous congestive heart failure | 86 (23.1%) | 98 (35.3%) | <0.001 |
| Atrial fibrillation | 179 (48.1%) | 108 (38.8%) | 0.019 |
| Previous renal dysfunction | 26 (7.0%) | 82 (29.5%) | <0.001 |
| Cerebral infarction | 54 (14.5%) | 56 (20.1%) | 0.058 |
| Cancer | 28 (7.5%) | 30 (10.8%) | 0.149 |
| Cirrhosis | 3 (0.8%) | 4 (1.4%) | 0.468 |
| NYHA classification |  |  | 0.059 |
| 2 | 105 (28.2%) | 62 (22.3%) |  |
| 3 | 174 (46.8%) | 125 (45.0%) |  |
| 4 | 93 (25.0%) | 91 (32.7%) |  |
| Heart rate (beats/min) | 92.0 (22.3) | 91.4 (24.5) | 0.390 |
| Systolic blood pressure (mmHg) | 130.0 (23.9) | 135.2 (25.7) | 0.019 |
| Diastolic blood pressure (mmHg) | 74.5 (16.7) | 73.9 (17.4) | 0.564 |
| Rales (>1/2 lung fields) | 158 (42.5%) | 153 (55.0%) | 0.002 |
| Jugular venous distension | 59 (15.9%) | 72 (25.9%) | 0.002 |
| Peripheral edema | 223 (59.9%) | 186 (66.9%) | 0.069 |
| LVEF (%) | 50.9 (12.6) | 51.2 (11.7) | 0.844 |
| B-type natriuretic peptide (pg/ml) | 637.5 (319.8-1245.3) | 1125.0 (472.8-2221.5) | <0.001 |
| Hemoglobin (g/L) | 122.1 (24.7) | 111.9 (23.1) | <0.001 |
| Hematocrit (%) | 0.4 (0.1) | 0.3 (0.1) | <0.001 |
| C-reactive protein (mg/L) | 9.4 (3.8-24.3) | 9.1 (4.5-25.0) | 0.538 |
| Alanine aminotransferase (IU/L) | 17.8 (12.1-31.6) | 14.7 (10.3-22.8) | <0.001 |
| Total bilirubin | 15.0 (10.5-22.8) | 13.0 (9.0-20.1) | 0.003 |
| Blood urea nitrogen (mmol/L) | 7.4 (5.7-9.7) | 9.8 (6.8-14.6) | <0.001 |
| Creatinine (μmol/L) | 84.3 (69.7-104.9) | 115.7 (84.7-149.2) | <0.001 |
| Albumin (g/L) | 36.7 (4.5) | 34.9 (4.4) | <0.001 |
| Sodium (mmol/L) | 138.5 (5.5) | 138.0 (5.1) | 0.188 |
| Potassium (mmol/L) | 4.2 (0.6) | 4.3 (0.7) | 0.248 |
| Uric acid (μmol/L) | 400.5 (300.1-511.0) | 405.1 (325.2-543.5) | 0.135 |
| Glucose (mmol/L) | 8.1 (3.5) | 8.9 (4.4) | 0.041 |
| Aldosterone antagonists | 298 (80.1%) | 222 (79.9%) | 0.937 |
| Loop diuretic | 353 (94.9%) | 270 (97.1%) | 0.159 |
| ACE-Is/ARBs | 166 (44.6%) | 93 (33.5%) | 0.004 |
| Beta-blockers | 276 (74.2%) | 198 (71.2%) | 0.399 |
| Anticoagulants | 138 (37.1%) | 83 (29.9%) | 0.054 |
| Aspirin | 151 (40.6%) | 120 (43.2%) | 0.510 |
| NSAIDs | 16 (4.3%) | 22 (7.9%) | 0.052 |
| Vasopressor | 35 (9.4%) | 25 (9.0%) | 0.856 |
| IABP | 4 (1.1%) | 6 (2.2%) | 0.339 |
| Mechanical ventilator | 62 (16.7%) | 66 (23.7%) | 0.025 |

P-value: The continuous data and category data between the AKI and non-AKI groups were compared by Kruskal Wallis rank sum test and Fisher's exact probability test, respectively.

Abbreviations:

NYHA, New York Heart Association

LVEF, left ventricular ejection fraction

ACE-Is/ARBs, angiotensin-converting enzyme inhibitors / Angiotensin Receptor Blockers

NSAIDs, Non-Steroidal Antiinflammatory Drugs

IABP, intra-aortic ballon pump

Table S4. Variables and regression coefficients of the four models.

| Model | A | B | C | D |
| --- | --- | --- | --- | --- |
| Approache | Stepwise | MFP | LASSO  lambda.1se | LASSO  lambda.min |
| The number of variables | 15 | 15 | 7 | 19 |
| (Intercept) | -2.9467 | -3.7260 | -2.7271 | -3.2665 |
| Age（years） | 0.0487 | 4.7277 | 0.0396 | 0.0467 |
| Diabetes | 0.3602 | 0.4519 | 0.4773 | 0.3420 |
| Atrial fibrillation | -0.3157 | -0.3030 |  | -0.3074 |
| Previous renal dysfunction | 0.7736 | 0.8179 | 0.6616 | 0.7092 |
| Cerebral infarction |  | 0.2839 |  | 0.3277 |
| Cirrhosis | 1.2651 | 1.2324 |  | 1.0655 |
| Systolic blood pressure (mmHg) |  |  |  | 0.0032 |
| Rales (>1/2 lung fields) | 0.2958 | 0.2452 |  | 0.1642 |
| Peripheral edema | -0.3454 | -0.3399 |  |  |
| B-type natriuretic peptide (pg/ml) | 0.0003 | 0.2556 | 0.0003 | 0.0002 |
| Hematocrit (%) |  |  | -0.9083 | -1.0349 |
| C-reactive protein (mg/L) | -0.0075 | -0.0663 |  | -0.0069 |
| Alanine aminotransferase (IU/L) |  |  |  | 0.0012 |
| Blood urea nitrogen (mmol/L) |  |  |  |  |
| Creatinine (μmol/L) | 0.0104 | 0.9030 | 0.0084 | 0.0105 |
| Albumin (g/L) | -0.0656 | -7.2319 | -0.0519 | -0.0577 |
| Potassium (mmol/L) | -0.3340 |  |  | -0.3554 |
| Glucose (mmol/L) | 0.0477 |  |  | 0.0470 |
| Aldosterone antagonists |  | 0.3245 |  | 0.3068 |
| Loop diuretic | 0.9052 | 0.7793 |  | 0.6513 |
| NSAIDs | 0.9562 | 1.0105 |  | 0.9217 |

Create new variables for MFP: B-type natriuretic peptide divided by 1000, age, creatinine, albumin divided by 100, C-reactive protein divided by 10.

Abbreviations:

MFP, multifractional polynomial

LASSO, least absolute shrinkage and selection operator

NSAIDs, Non-Steroidal Antiinflammatory Drugs

Table S5 Comparison of the four models

| Model | A | B | C | D |
| --- | --- | --- | --- | --- |
| Approache | Stepwise | MFP | LASSO  lambda.1se | LASSO  lambda.min |
| C-Statistics | 0.7904 | 0.7866 | 0.756 | 0.5379 |
| 95%CI low | 0.7553 | 0.7514 | 0.7184 | 0.4931 |
| 95%CI upp | 0.8255 | 0.8219 | 0.7936 | 0.5828 |
| Best threshold | -0.6511 | -0.4575 | -0.3825 | 21.7316 |
| Specificity | 0.6371 | 0.7016 | 0.707 | 0.5376 |
| Sensitivity | 0.8165 | 0.759 | 0.723 | 0.536 |

Abbreviations:

MFP, multifractional polynomial

LASSO, least absolute shrinkage and selection operator

Table S6. The regression coefficient of the model

|  | Estimate | Std error | OR | 95%CI.low | 95%CI.upp | P-value |
| --- | --- | --- | --- | --- | --- | --- |
| (Intercept) | -3.0249 | 1.0732 | 0.0486 | 0.0059 | 0.3979 | 0.0048 |
| Age | 0.0410 | 0.0082 | 1.0418 | 1.0253 | 1.0587 | 0.0000 |
| Diabetes | 0.4803 | 0.1836 | 1.6166 | 1.1280 | 2.3168 | 0.0089 |
| Previous renal dysfunction | 0.6950 | 0.2873 | 2.0037 | 1.1410 | 3.5185 | 0.0156 |
| Creatinine | 0.0085 | 0.0020 | 1.0085 | 1.0047 | 1.0125 | 0.0000 |
| B-type natriuretic peptide | 0.0003 | 0.0001 | 1.0003 | 1.0001 | 1.0004 | 0.0004 |
| Albumin | -0.0560 | 0.0207 | 0.9455 | 0.9080 | 0.9847 | 0.0068 |


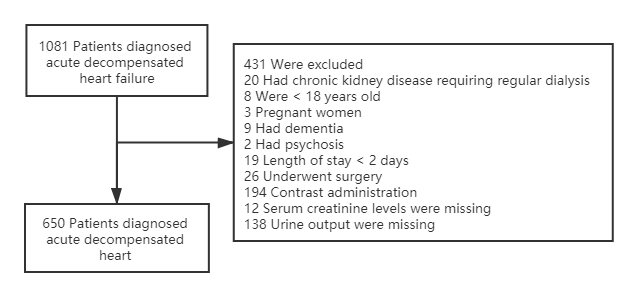


Figure. S1 Flow chart demonstrating the process of patient screening of a 1081 patients’ sample


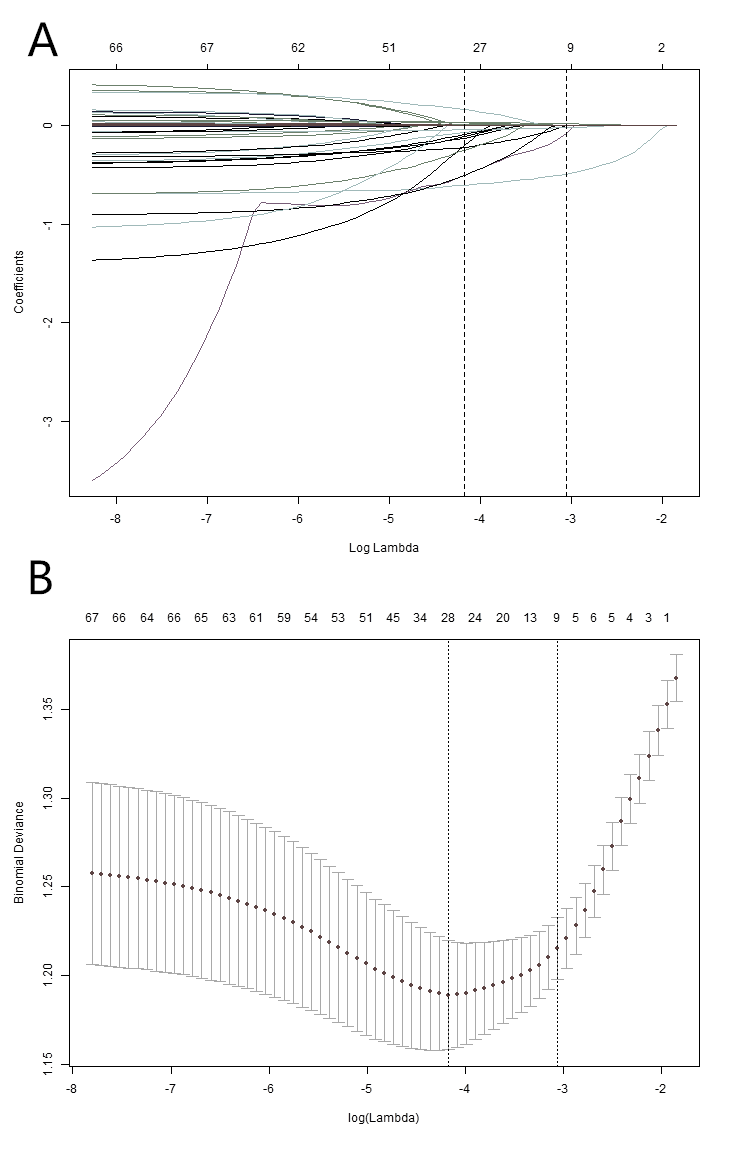


Figure S2 LASSO model profile plots. A, Tuning parameter (lambda: λ) selection in the LASSO model used 10-fold cross-validation via minimum criteria. B, LASSO coefficient profiles of the features against the log(λ).

Abbreviations:

LASSO, least absolute shrinkage and selection operator.


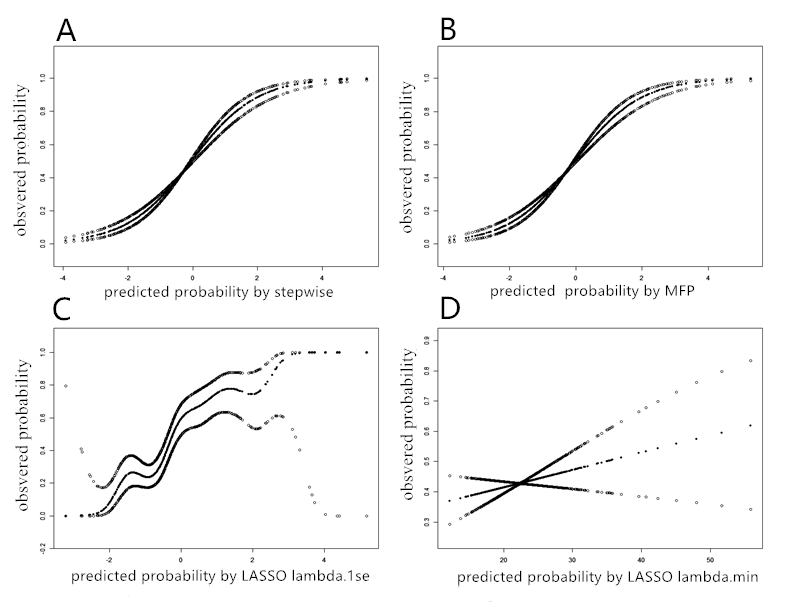


Figure S3 Calibration curve of each model

Calibration plots by stepwise (A), multifractional polynomial (MFP) (B), least absolute shrinkage and selection operator (LASSO) regression (model C and model D according to tuning parameter lambda.1se and lambda.min respectively)


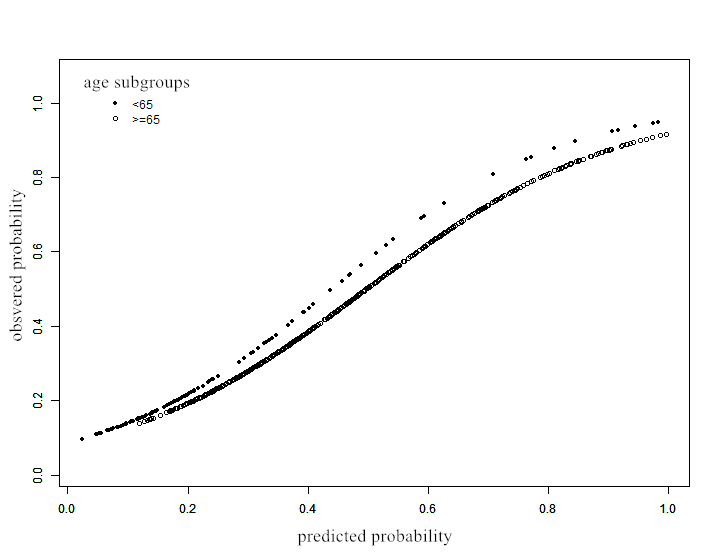


Figure S4 The calibration curve of the prediction model in older and younger subgroups.
